# Social and environmental conditions related to *Mycobacterium leprae* infection in children and adolescents from three leprosy endemic regions of Colombia

**DOI:** 10.1186/s12879-019-4120-2

**Published:** 2019-06-13

**Authors:** Héctor Serrano-Coll, Hugo Rene Mora, Juan Camilo Beltrán, Malcolm S. Duthie, Nora Cardona-Castro

**Affiliations:** 1 Graduate School - Universidad CES, Medellín, Colombia; 2 Instituto Colombiano de Medicina Tropical-Universidad CES, Sabaneta, Colombia; 3 Infectious Disease Research Institute, Seattle, USA; 4 Instituto Colombiano de Medicina Tropical, Faculty of Medicine, Escuela de Graduados – Universidad CES, Cra 43 A # 52 Sur 99., Sabaneta, Colombia

**Keywords:** Leprosy, Antibodies, Children, Adolescents, *Mycobacterium leprae*

## Abstract

**Background:**

Leprosy is is still considered a public health issue and in Colombia 7–10% of new cases are found in children, indicating both active transmission and social inequality. We hypothesized that circulating antibodies against Natural Octyl Disaccharide-Leprosy IDRI Diagnostic (NDO-LID) (a combination of *Mycobacterium leprae* antigens) could reveal the social and environmental aspects associated with higher frequencies of *M*. *leprae* infection among children and adolescents in Colombia.

**Methods:**

An observational cross-sectional study was conducted involving sampling from 82 children and adolescents (younger than 18 years of age) who had household contact with index leprosy patients diagnosed in the last 5 years. Data were analyzed through bivariate analysis made by applying a Pearson x^2^ test for qualitative variables, while quantitative variables, depending on their distribution, were analyzed using either a Student’s t-test or Mann-Whitney U test. Multivariate analysis was performed using a multiple regression and binomial logistic approach.

**Results:**

A bivariate analysis demonstrated that antibody titers against NDO-LID were significantly greater in children and adolescents with a low socioeconomic status that had: lived in vulnerable areas of the UAChR shared region; eaten armadillo meat; exposure of over 10 years to an index case and; not received BCG immunization. Moreover, a multivariate analysis showed that residing in the UAChR region has a strong association with a greater possibility of *M. leprae* infection.

**Conclusions:**

*M. leprae* transmission persists among young Colombians, and this is associated with social and environmental conditions. An intensification of efforts to identify new leprosy cases in vulnerable and forgotten populations where *M. leprae* transmission continues therefore appears necessary.

**Electronic supplementary material:**

The online version of this article (10.1186/s12879-019-4120-2) contains supplementary material, which is available to authorized users.

## Background

Leprosy is a chronic infectious disease caused by *Mycobacterium leprae*, an alcohol-acid resistant bacillus that has a notable affinity for the skin and peripheral nerves [1]. From an epidemiological perspective, approximately 225,000 new leprosy cases are diagnosed each year around the world, with 8.9% of these cases occurring in children and adolescents [2, 3]. Furthermore, at the time of diagnosis 11% of these children already present with grade 2 disability increasing to 27.3% during their follow-up [3].

In Colombia, 300–500 new cases of leprosy are reported per year. Seven percent of the new leprosy cases occurred in children younger than 15 years of age, 59% of these cases were in children 10–14 years of age [4, 5]. Although there is a low prevalence of leprosy in Colombia as a whole (< 1/10000 inhabitants), we have detected high transmission of *M. leprae* with certain at risk populations [5]. Thorough evaluation by physical examination, detection of antibodies against the gold standard antigen phenolic glycolipid (PGL)-I, and PCR detection of *M. leprae* DNA in nasal swabs and suspicious skin lesions have contributed to the detection of both new patients and infected but healthy household contacts (HHC). In addition, molecular epidemiology studies in Colombia have demonstrated intra-familiar *M. leprae* transmission [6]. Determining who is infected with *M. leprae* is complicated by the inability to isolate and culture the bacteria ex vivo. Antibodies against *M. leprae* are therefore considered as a simple proxy indicator of infection or, at a minimum, exposure. We previously reported that the synthetic mimetic of PGL-I, natural octyl disaccharide (NDO), can be combined with recombinant protein Leprosy IDRI Diagnostic (LID)-1 to provide the NDO-LID conjugate that can detect antibodies in the serum of leprosy patients and many HHC [7]. In patients, the magnitude of the antibody response correlates strongly with the level of infection [7].

Leprosy among children and adolescents has been correlated with certain social and environmental aspects, such as cohabiting with an index case, malnutrition, living in overcrowded households, and absence of bacillus Calmette-Guerin (BCG) immunization scars [4, 8]. Likewise, *M. leprae* infection could also be linked to interactions with potential animal reservoirs such as armadillos [9]. Importantly, as it was mentioned, 7% of Colombian cases occur in children younger than 15 years of age with approximately 3 of every 5 of these cases are in children aged 10–14 years old [4, 5]. These data are alarming because they reveal ongoing and active transmission of *M. leprae* that, on top of a diagnostic lag, indicate shortcomings in the elimination efforts of leprosy control programs.

Given that detection of serum antibodies against *M. leprae* is more practical among at risk populations than direct detection methods (PCR and histopathology) [6], we hypothesized that these responses used in the context of various socioeconomic indicators could enhance our understanding of factors that contribute to the risk of *M. leprae* infection and development of leprosy. The objective of this study was therefore to determine the relationship between anti-NDO-LID antibodies (IgM, IgG, protein A titers (i.e. antibodies for both IgM and IgG)), taking into account that the levels of these immunoglobulins are strongly linked with *M. leprae* infection levels [7, 10], and social and environmental aspects that could be associated with a higher *M. leprae* infection rate in children and adolescents in the higher risk regions of Colombia.

## Methods

### Study and sample description

An observational cross-sectional study was conducted during 2015 and 2016 with a sample frame made of 82 selected children and adolescents 18 years and younger. Each had HHC with a leprosy patient that had been diagnosed in the last 5 years, and all were included for convenience sampling. The study population was derived from three geographical regions where the reporting of new leprosy cases is greater than other parts of Colombia: Uraba-Antioquia-Choco (UAChR; *n* = 18 children), from the Caribbean Region (CR; *n* = 43), and from the Andean Region (AR; *n* = 21).

### Sociodemographic and environmental characteristics compilation

Sociodemographic characteristics were gathered using a HHC evaluation form implemented by the leprosy research team of the Instituto Colombiano de Medicina Tropical (ICMT). For the purposes of this study we focussed on: age, sex, socioeconomic status, geographic location, recorded armadillo consumption, BCG immunization status, and clinical form of the index case. The socioeconomic categories established by the Colombian government are named status 1. Low-low, 2.Low, 3. Medium-low, 4. Medium, 5. Medium-high, 6. High. Status 1, 2 and 3 corresponding to low status that included the people of scarce resources [11].

### Measurement of antigen-specific serum antibodies

Serum samples were collected for subsequent serologic evaluation. Briefly each well of 96-well ELISA plates (Nunc-Immuno 96-well, Polysorp plates) was coated with 1 μg/ml NDO-LID antigen at room temperature then blocked using 100 μl blocking buffer (1% bovine serum albumin, BSA/ phosphate-buffered saline, PBS/ PBS + Tween20, PBS-T). Plates were incubated for 1 h with agitation at room temperature. Plates were washed (5 PBS-T + 2 PBS), and 50 μl serum (1:200 dilution in BSA 0.1%/ PBS/ PBS-T) was added, followed by a 1 h of incubation with agitation at room temperature. Subsequently, 50 μl horseradish peroxidase (HRP)-conjugated detector diluted in BSA 0.1%/ PBS/ PBS-T was added and plates incubated for 1 h with agitation at room temperature. Three detectors were evaluated: anti-human IgG, anti-human IgM and protein A (Rockland Immunochemicals Inc., Limerick, PA, USA). After incubation and washing, 50 μl TMB substrate was added for 15 min in the dark at room temperature, then stopped by adding 25 μl 1 N sulfuric acid. Optical densities (OD) were measured at 450 nm using an ELISA plate reader (Spectrophotometer Bio-Rad Xmark) [10]. Cut-off values were assessed as the average OD plus two standard deviations obtained from sera (*n* = 100) of healthy individuals that resided in an area not endemic for leprosy. Cut-off values of 0.127, 0.226 and 0.183 were obtained for Protein A, IgM and IgG, respectively.

### Statistical analyses

Data was analyzed using Excel and SPSS 24.0 software. Univariate analysis of qualitative variables was made via absolute and relative frequencies calculation. For quantitative variables summary measures like central tendency were performed. The distribution of variables was obtained using a Kolmogorov-Smirnov test. Bivariate analysis between qualitative variables was made by applying a Pearson x^2^ test and quantitative variables were analyzed using a Student’s t-test or Mann-Whitney U test, taking into account the distribution of these variables. Multivariate analysis was conducted using a multiple regression and binomial logistic approach. Significance level of *p*-value < 0.05 were established for all analysis and a risk approximation was made using an odds ratio calculation (OR) with its own confidence interval (95%CI).

### Ethics declaration

This study was considered of minimal risk and was approved by the Instituto Colombiano de Medicina Tropical – Universidad CES ethics committee. After the aims of the study were explained, an informed consent form was signed by the guardians of participating children.

## Results

### Sample characterization

A total of 82 children and adolescents were included, of which 36 (44%) were male and 46 (56%) were female. The age range was 1 to 18 years of age, with a mean of 10.7 years (Table 1). Of note, the majority of these children and adolescents lived with an index leprosy case: 57 with a lepromatous case; 12 with a pure neural leprosy case, 10 with a dimorphic leprosy case, 2 with an intermediate leprosy case and 1 with a tuberculoid case.

**Table 1:** Sample characteristics of the study cohort

Index case of leprosy	Sample size N (%) HHC	Sex, M/F N (%)	Average age (range), years
I	2 (2%)	2 (100%M)	9 (4–14)
TT	1 (1%)	1 (100%F)	11
D	10 (12%)	6/4 (60–40%)	12.4 (5–17)
LL	57 (70%)	23/34 (40–60%)	10.1 (1–18)
NL	12 (15%)	8/4 (67–34%)	13.1(7–18)
Total	82	36/46 (44 /56%)	10.7 (1–18)

*M* male, *F* female, *HHC* household contact, *I* indeterminate leprosy, *TT* tuberculoid leprosy, *D* dimorphic leprosy, *LL* lepromatous leprosy, *NL* neural leprosy, *N* number

### Relationship between antibody responses and socioeconomic status

In accordance with the socioeconomic categories established by the Colombian government, we found that 42 (51%) of the children and adolescents could be considered of low-low socioeconomic status while 40 (49%) could be considered of low or a medium-low status. When seropositivity against NDO-LID was compared in these two sample groups, the frequency of anti-NDO-LID antibodies detected by Protein A (i.e. of both IgG and IgM isotypes) and IgM was highest among children of lower socioeconomic backgrounds (Table 2;*p*-value =0.001).

**Table 2:** Differences of NDO-LID antibodies according to their socioeconomic status and origin region

Variable	Sample size	Anti-NDO-LID			Anti-NDO-LID IgM			Anti- NDO-LID IgG		
	N (%)	protein A								
		(+)	OR (95% CI)	*P*-value	(+)	OR (95% CI)	*P*-value	(+)	OR (95% CI)	*P*-value
		N (%)			N (%)			N (%)		
SEC Low-low	42 (51)	15 (36)	10.55 (2.2–52.5)	0.001	18 (43)	6.75 (2–22.4)	0.001	5 (12)	5.3 (0.59–47.2)	0.102
SEC Low or Medium-low	40 (49)	2 (5)			4 (10)			1 (3)		
Origin UAChR	18 (22)	12 (67)	3.9 (1.9–8.1) UAChR vs AR	< 0.0001	15 (83)	7.5 (1.98–28.3) UAChR vs AR	< 0.0001	3 (6)	2.5 (1.6–3.7) UAChR vs AR	0.136
Origin CR	43 (52)	4 (9)			3 (7)			3 (7)		
Origin AR	21 (26)	1 (5)	5.6 (2.5–12.4) UAChR vs CR		4 (19)	11.9 (3.9–36.2) UAChR vs CR		0	1.8 (0.7–4.5) UAChR vs CR	

*OR* odds ratio, *CI* confidence interval, (+) positive for the presence of anti-NDO-LID antibodies, *SEC* socioeconomic status, *UAChR* Uraba-Antioquia-Choco shared region, *CR* Caribbean region, *AR* Andean region, *OR* odds ratio, *CI* confidence interval, (+) positive for the presence of anti-NDO-LID antibodies

**Fig. 1:**
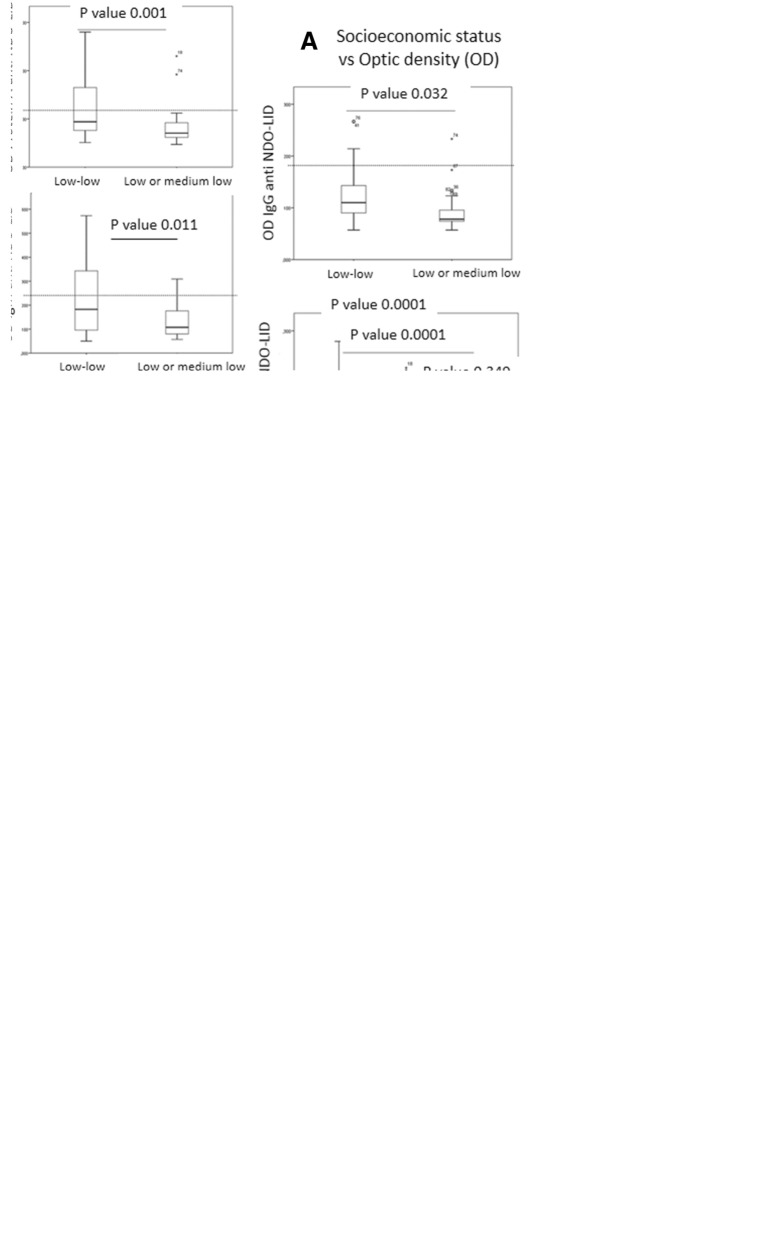
Impact of socioeconomic and geography on *M. leprae* levels. Anti-natural octyl disaccharide-leprosy IDRI diagnostic (NDO-LID) antibody levels in children and adolescents were measured by ELISA, using either protein A, anti- IgM or anti-IgG to detect responses. In **a**, samples were stratified by socioeconomic status as either low-low (*n* = 42) or low or medium-low (*n* = 40). In **b**, samples were stratified by geographic source as UAChR (*n* = 18), Caribbean Region (*n* = 43), or Andean Region (*n* = 21). In **c**, samples from the low-low socioeconomic group were subdivided by geographic source as UAChR (*n* = 41), Caribbean Region (*n* = 43), or Andean Region (*n* = 17). Data are displayed as box and whisker plots, with the box representing the Q1 to Q3 interquartile range and the horizontal bar representing the median of the optical density of the samples. Individual dots indicate outliers, and *p*-values are indicated by the lines above each indicated group

In addition to the simple presence of antibodies, we determined anti-NDO-LID antibody levels. Significantly stronger anti-NDO-LID Protein A (*p* = 0.001), IgM (*p* = 0.011) and IgG (*p* = 0.032) responses were detected in children and adolescents of low-low status relative to those observed in samples from subjects of low or medium-low status (Fig. 1a). These data imply that, in addition to a greater rate of *M. leprae* infection, the overall levels of infection in each child or adolescent of low-low socioeconomic status are higher.

### Relationship between antibody responses and geographic region

When assessing anti-NDO-LID antibodies detected by protein A and IgM according to the geographic origin of subjects, we found higher frequency of seropositivity in children and adolescents from UAChR (*P* = 0.0001, see Table 2).

Antibody titers were also compared based on the geographic origin of the subject. We found that anti-NDO-LID responses detected by Protein A, IgM and IgG in children and adolescents from UAChR region were significantly greater (all *p*-values = 0.0001) than the levels measured among samples from the Caribbean and Andean regions (Fig. 1b). Taken together, these results suggest that children and adolescents from UAChR are under higher pressure for *M.* leprae infection than those that reside around the Caribbean or in the Andes.

### Antibody responses among children and adolescents of low-low socioeconomic status according to their geographic origin

Based on observations made on socioeconomic status in each region of study, we noticed that low-low, low and medium-low status differ according to the development level of each region. We therefore decided to compare seropositivity of children and adolescents belonging to a low-low socioeconomic status for each geographic area studied (Table 3). Subsequently, comparison of seropositivity rates by low-low status for each regions revealed a higher seropositivity for IgM and protein A in the UAChR compared with the seropositivity rates found in the Caribbean and the Andes (*p*-value < 0.05). Significant differences in the magnitude of antibody responses of low-low status individuals were also observed across the regions. When compared to Andean and Caribbean population, significantly higher levels of anti-NDO-LID antibodies were detected in individuals belonging to low-low socioeconomic status that resided in UAChR (Fig. 1c). This data further implies that a higher *M. leprae* frequency of infection occurs in children and adolescents with social inequalities found in a determined geographical region.

**Table 3:** Differences of NDO-LID antibodies according to low- low socioeconomic status and geographic area

Low-low socioeconomic status according to the geographic area	Sample size N (%)	Anti-NDO-LID protein A			Anti-NDO-LID IgM			Anti- NDO-LID IgG		
		(+) N (%)	OR (95% CI)	*P*-value	(+) N (%)	OR (95% CI)	*P*-value	(+) N (%)	OR (95% CI)	*P*-value
Low-low- UAChR	17 (41)	12 (71)	3.6 (1.6–7.9) UAChR vs CR	0.001	15 (88)	8.9 (2.4–33.2) UAChR vs CR	< 0.0001	3 (18)	2.5 (1.6–3.7) UAChR vs CR	0.474
Low-low - CR	18 (43)	2 (11)			1 (6)			2 (11)		
Low-low- AR	7 (17)	1 (14)	2 (1.04–3.9) UAChR vs AR		2 (29)	3.1 (1–11) UAChR vs AR		0	1.5 (1.2–2) UAChR vs AR	

*UAChR* Uraba-Antioquia-Choco shared region, *CR* Caribbean region, *AR* Andean region, *OR* odds ratio, *CI* confidence interval, (+) positive for the presence of anti-NDO-LID antibodies

### Impact of armadillo consumption on serologic responses

**Fig. 2:**
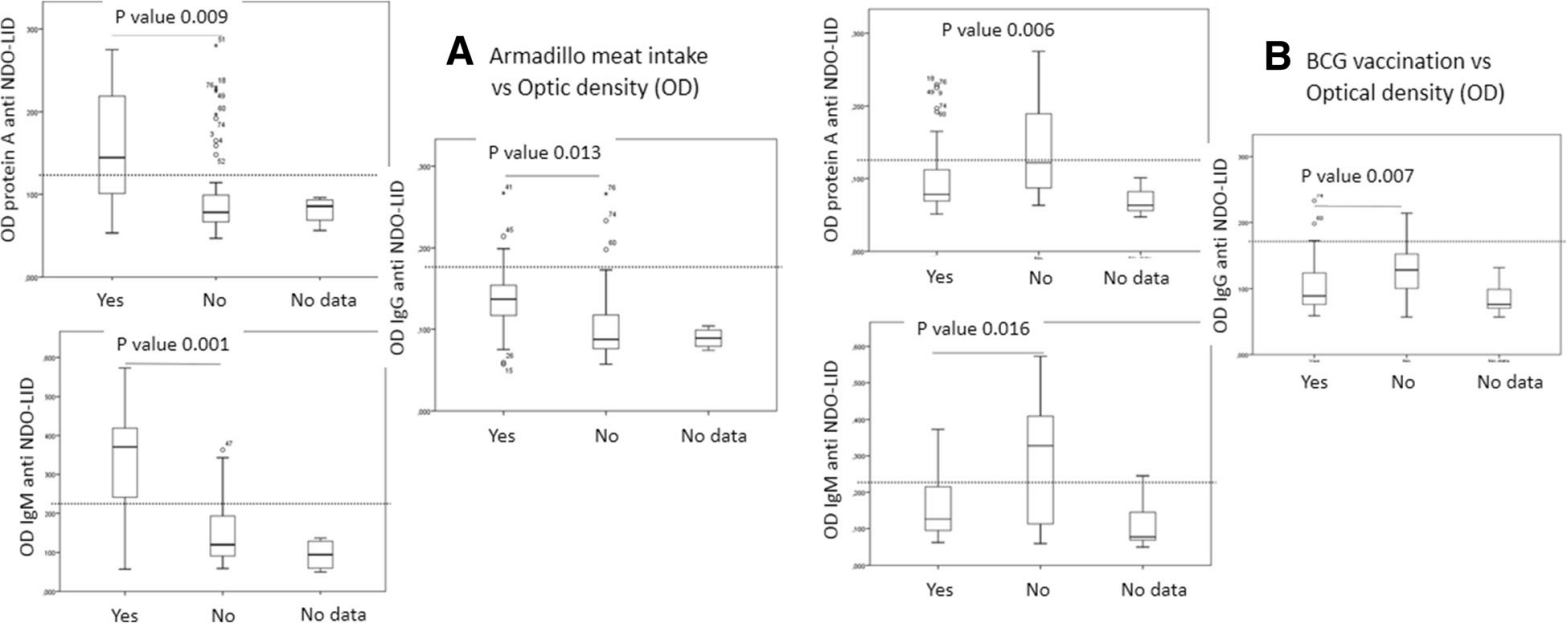
Impact of behavioral variables on *M. leprae* infection levels. Anti-natural octyl disaccharide-leprosy IDRI diagnostic (NDO-LID) antibody levels in children and adolescents were measured by ELISA, using either protein A, anti- IgM or anti-IgG to detect responses. In **a**, samples were stratified by recorded knowledge of eating armadillo meat as either yes (*n* = 14) or no (*n* = 64). In **b**, samples were stratified by recorded knowledge of BCG re-vaccination following identification of the index leprosy case as either yes (*n* = 54) or no (*n* = 16). Data are displayed as box and whisker plots, with the box representing the Q1 to Q3 interquartile range and the horizontal bar representing the median of the optical density of the samples. Individual dots indicate outliers, and *p*-values are indicated by the lines above each indicated group

Upon questioning, we assessed that 14 individuals reported consumption of armadillo meat at some point (17.1% of the overall cohort, although for 4 (4.9%) this data was not documented). We found that anti-NDO-LID antibodies could be detected by Protein A in 9 of 14 (64%) armadillo consumers in contrast to only 8 of 64 (12%) in those that had not eaten armadillo meat (Table 4, *p*-value < 0.0001). Furthermore, overall anti-NDO-LID antibody levels were higher in children and adolescents that had consumed armadillo meat compared to those that had not (*p*-value = 0.009 for Protein A, 0.001 for IgM and 0.013 for IgG) (Fig. 2a). These findings indicate that armadillo consumption is strongly associated with both the rate and level of anti-NDO-LID antibodies.

**Table 4:** Differences of NDO-LID antibodies according to armadillo meat intake, BCG re-vaccination, and time of exposure

Variable	N (%)	Anti-NDO-LID protein A			Anti-NDO-LID IgM			Anti- NDO-LID IgG		
		(+)	OR (95% CI)	*P*-value	(+)	OR (95% CI)	*P*-value	(+)	OR (95% CI)	*P*-value
		N (%)			N (%)			N (%)		
Armadillo meat intake	14 (17)	9 (64)	12.6 (3.3–47)	< 0.0001	11 (79)	17.6 (4.2–74)	< 0.0001	3 (21)	5.5 (0.9–31)	0.79
Non armadillo meat intake	64 (78)	8 (12)			11 (17)			3 (5)		
BCG re-vaccination	54 (66)	9 (17)	0.2 (0.059–0.67)	0.002	12 (22)	0.22 (−0.07–0.72)	0.008	3 (6)	0.25 (0.046–1.4)	0.118
No BCG re-vaccination	16 (19)	8 (50)			9 (56)			3 (19)		
Time of exposure < 10 years	45 (55)	6 (13)	1.6 (1.02–2.56)	0.068	8 (18)	1.6 (1.05–2.6)	0.041	1 (2)	1.9 (1.2–3)	0.086
Time of exposure > 10 years	37 (45)	11 (30)			14 (38)			5 (14)		

*OR* odds ratio, *CI* confidence interval, (+) positive for the presence of anti-NDO-LID antibodies

### Impact of BCG re-vaccination status

A recommendation within leprosy control programs has been the secondary (or initial) BCG vaccination for contacts of diagnosed index cases. Accordingly, we found that 54 (66%) of evaluated children and adolescents had received reinforcement with BCG after their index case had been diagnosed, whereas 16 (19%) had not received this reinforcement (data was not documented in 12 (15%) of the study cohort). The frequency of anti-NDO-LID antibodies was higher (Table 4, *p*-value < 0.01), and elevated titers for those detected by Protein A (*p*-value =0.006), IgM (*p*-value = 0.016) and IgG (*p*-value = 0.007), were observed in individuals that had received reinforcement with BCG (Fig. 2b). Thus, a positive BCG immunization status was related to both lower rates and levels of *M. leprae* infection but lack of BCG presented as a risk.

### Relationship of serum antibody responses of children with the clinical form of their index case

**Fig. 3:**
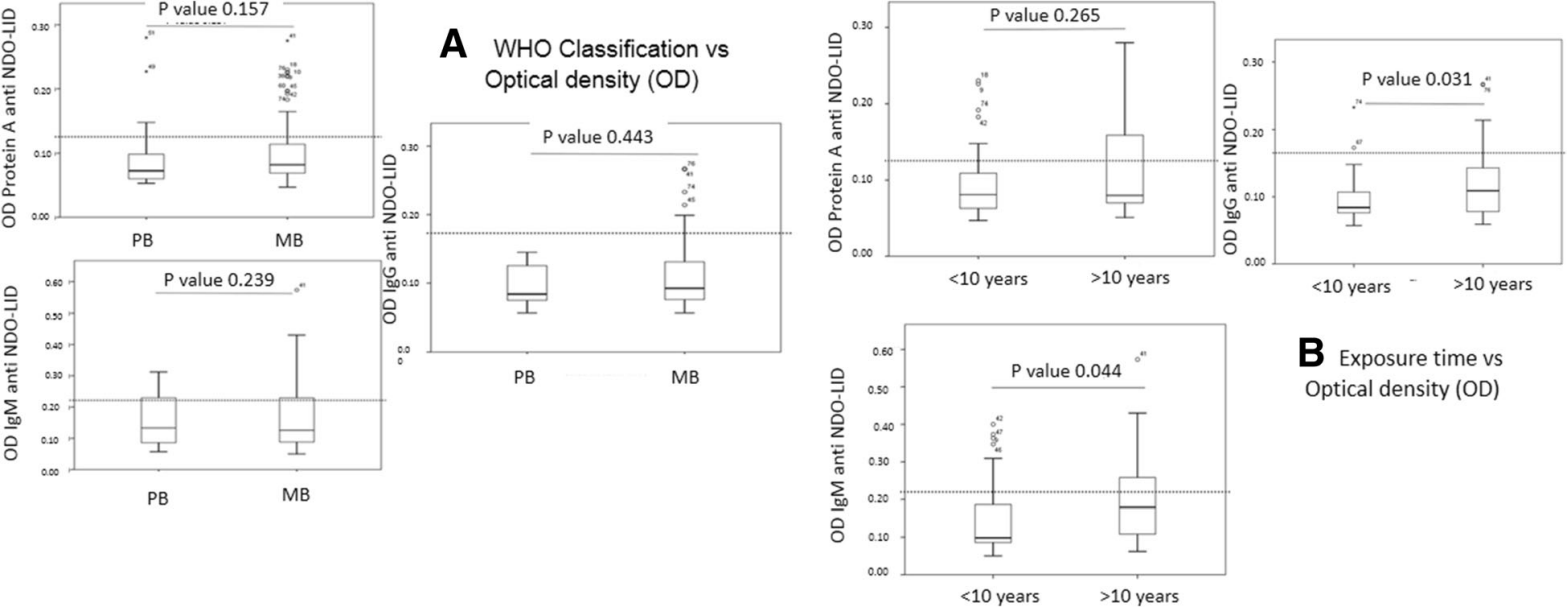
Influence of index case on *M. leprae* infection levels. Anti-natural octyl disaccharide-leprosy IDRI diagnostic (NDO-LID) antibody levels in children and adolescents were measured by ELISA, using either protein A, anti- IgM or anti-IgG to detect responses. In **a**, samples were stratified by reported WHO operational classification of the index case as either MB (*n* = 66) or PB (*n* = 16). In **b**, samples were stratified by estimated duration of exposure to the index leprosy case as either less than 10 years (*n* = 45) or greater than 10 years (*n* = 37). Data are displayed as box and whisker plots, with the box representing the Q1 to Q3 interquartile range and the horizontal bar representing the median of the optical density of the samples. Individual dots indicate outliers, and *p*-values are indicated by the lines above each indicated group

It is well documented that contacts of multibacillary (MB) cases are at elevated risk of developing leprosy. We found that the majority (66; 80.5%) of the study subjects lived with a MB index case while 16 (19.5%) lived with a paucibacillary (PB) index case. Surprisingly, anti-NDO-LID antibodies detected by Protein A, IgM or IgG showed similar rates between MB or PB cases (*p*-value > 0.05). Similarly, differences were not observed in the overall antibody levels between children living with either MB or PB cases (*p*-value > 0.05, Fig. 3a). Therefore, in our evaluations the antibody responses of the children did not appear to be influenced by the clinical form of their respective index case.

### Relation of antibody responses with the exposure time to the leprosy index case

Although the frequency of *M. leprae* infection was not impacted to the clinical form of the leprosy index case, we queried if the length of exposure to the leprosy index case could have an impact. Forty-five (54.9%) children and adolescents reported an exposure of less than 10 years while 37 (45.1%) reported exposure periods longer than 10 years (Table 4). When analyzing the presence of circulating antibodies, we found that anti-NDO-LID IgM levels were higher in individuals with > 10 years of exposure *p*-value = 0.044) (Fig. 3b). These data support the hypothesis that prolonged exposure to a leprosy index case increases the risk of *M. leprae* infection.

### Relation of antibody responses with evaluated social and environmental variables

A binomial logistic analysis indicated that the single variable that most strongly associated with the frequency of *M. leprae* infection and increase antibody titers in children and adolescents in Colombia was residency in a geographic region linked to extreme poverty, such as the UAChR shared region (IgM/P- value = 0.004, Protein A/*P*-value = 0.045). Furthermore, it is probable that consumption of armadillo meat, and prolonged exposure to a leprosy case can be related with increase the risk to infection to *M. leprae* (Table 5).

**Table 5:** Differences NDO-LID seropositivity according to social and environmental factors evaluated

Factors	IgM Anti-NDO-LID (ExpB)	95% CI	*P*-value	Protein A Anti NDO-LID (ExpB)	95% CI	*P*-value	IgG Anti NDO-LID (ExpB)	95% CI	*P*-value
Geographic area	19.8	2.6–150	0.004	6.5	1.04–41	0.045	0.678	0.3–13	0.8
Time of exposure	0.186	0.03–1.1	0.063	0.32	0.068–1.55	0.16	0.18	0.02–1.8	0.15
Armadillo meat intake	9.4	0.97–90	0.053	8.1	0.92–70	0.059	7.4	0.32–170	0.21
BCG re-vaccination	1.07	0.27–4.2	0.92	1.77	0.34–9.1	0.496	1.5	0.18–12	0.719
Socioeconomic status	1.87	0.35–9.8	0.462	3.3	0.48–22	0.22	3.9	0.26–58	0.32

*ExpB* Multivariate-adjusted Odds ratio, *CI* confidence interval

The assessed socio-environmental variables also corresponded with antibody titers when assessed by a multiple lineal analysis, confirming that these variables behave like predictors and do indeed correspond with anti-NDO-LID levels (Protein A (R = 0.59, R^2^ = 0.348, *p*-value = 0.0001), IgM (R = 0.746, R^2^ = 0.528, *p*-value =0.0001) and IgG (R = 0.518, R^2^ = 0.268, *p*-value = 0.0001).

The variables related to an increase in antibody titers in children and adolescents in the three endemic regions studied were: 1. residency in a geographic region linked to extreme poverty, such as the UAChR shared region (*p*-values < 0.0001 for IgM and 0.001 for Protein A); 2. > 10 years exposure to a leprosy case (*p*-values 0.018 for IgM and 0.027 for IgG); and 3. Consumption of armadillo meat (*p*-value 0.006 for IgM) (Table 6).

**Table 6:** Differences NDO-LID antibodies according to social and environmental factors evaluated

Factors	IgM Anti-NDO-LID		Protein A Anti-NDO-LID		IgG Anti- NDO-LID	
	OD^a^	*P*-value	OD^a^	*P*-value	OD^a^	*P*-value
Socioeconomic status - Geographic area - Armadillo meat intake- BCG re-vaccination- time of exposure	0.495^a^	< 0.0001	0.270^a^	< 0.0001	0.169^a^	< 0.0001
Geographic area	−1.42^b^	< 0.0001	−0.053^b^	0.001	−0.022^b^	0.115
Time of exposure	0.044^b^	0.018	–	–	0.021^b^	0.027
Armadillo meat intake	−0.064^b^	0.006	−0.02^b^	0.136	−0.016^b^	0.152
BCG re-vaccination	0.001^b^	0.940	−0.006^b^	0.384	0.003^b^	0.648
Socioeconomic status	−0.009^b^	0.651	−0.017^b^	0.160	−0.018	0.079

^a^OD: optic density average, ^b^change of OD anti-NDO-LID

These data allowed us to create a series of equations based on the algebraic formula of the multiple lineal regression model, to predict anti-NDO-LID antibody titers (implying *M. leprae* infection) in the children and adolescent populations of the Colombia regions studied. The collinearity of the model was tested using VIF (variance inflation factor), coefficient of determination R^2^, and residual analysis (Additional file 1). The model could be useful to predict IgM anti-NDO-LID antibody titers in the leprosy endemic regions evaluated. The model cannot be validated for Protein A and IgG anti NDO-LID.

### Algebraic formula of the multiple regression model

$$ Y={\beta}_0+{\beta}_1{x}_1+{\beta}_2{x}_2+\dots $$

Y: dependent variable, x: independent variable, β_0_: coefficient (constant) of the dependent variable, β_1_, β_2_: coefficient that signifies a change in the dependent variable when the independent variable is present.

**Predictive equation of anti-NDO-LID IgM titers =** 0.495 OD + [− 1.42 OD × 1 (not residing in a vulnerable geographic area) or × 0 (residing in a vulnerable geographic area)] + [0.044 OD × 1 (> 10 years exposure) or × 0 (< 10 years exposure)] + [− 0.064 OD × 1 (not consuming armadillo meat) or × 0 (consuming armadillo meat)].

## Discussion

Leprosy remains a public health problem in many areas. Establishing the relationship between *M. leprae* infection and socio-environmental factors may help identify why there is greater frequency of infection among juvenile HHC of leprosy patients relative to other contacts. Considering that leprosy has strong links to poverty [12, 13], we examined the rates and levels of serum suggestive of *M. leprae* infection against various indicators of reduced social status in Colombia. In agreement with the general findings of other, we determined that the lower the socioeconomic status of the household inhabited by children and adolescents, the higher rates and levels of antibodies against the diagnostic conjugate NDO-LID. Residency in a geographic region linked to extreme poverty and prolonged (> 10 years) exposure to a leprosy case were very influential, as was reported consumption of armadillo meat. Although limited to convenience sampling and a small sample collection that does not represent all Colombian regions, at a time when leprosy control program activities are generally being scaled back or integrated into general health systems, our data and methods could help focus control efforts, inform education campaigns and enhance the overall output of leprosy-specific programs.

It is widely reported and accepted that HHC of MB patients have a higher risk of *M. leprae* infection and subsequent development of leprosy than HHC of PB patients [14]. In contrast with the reported data of Amorim et al. [15], in which elevated antibody responses were found in HHC of MB patients compared to HHC of PB patients, our analyses found no significant differences in either anti-NDO-LID frequencies or levels when juvenile contacts were stratified by index case presentation. Although our previous data did not statistically relate serum antibody responses with duration of exposure to a patient [6] others have linked this with risk of *M. leprae* infection [15] and we therefore decided to assess this environmental variable and relationship among subgroups in the current study. Accordingly, we observed that individuals with > 10 years exposure had significantly greater rates of seropositivity than those with < 10 years exposure. These results suggest that those with a longer length of residency with a leprosy index case, and therefore greater cumulative exposure, have a greater likelihood of infection and of developing the disease.

Knowing that leprosy is associated with poverty is not particularly beneficial in focusing control efforts in Colombia because it has the third lowest distribution of wealth of any country in the world [16]. Our study indicates that the geographic region in which children and adolescents reside is, however, an important variable that presents risk for *M. leprae* infection. Serologic data from three distinct geographic regions (Uraba-Antioquia-Choco shared region, the Caribbean region, and the Andean region), revealed that juveniles in the UAChR region had a higher *M. leprae* infection rates than the other regions. These results can be explained in part by the inherent conditions of this region. UAChR is characterized for its poor public health conditions, being one of the most unequal distribution of wealth regions in Colombia, and for having parts of its territory lacking in fundamental public services like basic sanitation as well as electricity and water supply. This finding agrees with observations made in 139 municipalities in Tocantins, Brazil [13], where municipalities with a higher vulnerability and social inequality presented with increases in leprosy onset and spread of *M. leprae*.

When evaluating the relative impacts of either region of residence or socioeconomic status on *M. leprae* infection in the study populations, we found that, rather than belonging to a low-low socioeconomic status, inhabiting a vulnerable geographic region (without sanitary and public services like UAChR) was the key social variable that resulted in increased frequency of *M. leprae* infection. This was heightened by the fact that these vulnerable geographic regions have unfavorable socioeconomic conditions such as malnutrition, low schooling levels and absence of leprosy control program interventions [12, 17].

It is noteworthy to the region that *M. leprae* infection has been associated with armadillo hunting and manipulation [18, 19]. Given that leprosy can be considered somewhat zoonotic, that *M. leprae*-infected armadillos have been found in Colombia, and that armadillos are often used as a meat source, in traditional medicine and as pets, we hypothesized that armadillo consumption could be of great importance in the studied population [9, 20]. While Schmitt et al. [21] could not relate armadillo meat consumption with *M. leprae* infection in Brazil, arguing not only that cooking the meat would kill the mycobacterium but also that there is a lack of evidence for gastrointestinal transmission, we found that armadillo meat consumption correlated with higher frequencies of *M. leprae* infection in children and adolescents. This is consistent with other recent findings that armadillo meat consumption is related to a higher proportion of leprosy cases [19, 20].

Among the factors that appeared protective, BCG immunization was associated with reduced *M. leprae* infection frequencies among the children and adolescents in our study cohorts. The BCG vaccine can enhance cellular immune responses against *M. leprae*, and immunization with BCG has been recommended for HHC of diagnosed leprosy index cases [22]. Our findings are therefore compatible with both our previous research as well studies in Brazilian populations. As a pre-emptive approach to reduce the emergence of new cases and enable *M. leprae* elimination, it has recently been suggested that seropositive individuals that have asymptomatic or sub-clinical *M. leprae* infection receive BCG re-vaccination as well as treatment with rifampicin to serve as an early intervention to alter the natural course of infection [23, 24]. Our data provide further evidence for the implementation of prophylactic measures within high risk juvenile and other HHC populations.

In summary, our assessment among Colombian children and adolescents in contact with leprosy cases reveals that risk of *M. leprae* infection is increased by: residency in a vulnerable geographic region; > 10 years exposure to the leprosy index case; and consumption of armadillo meat. These results are similar to those obtained in other socioeconomic analyses [12, 13, 18] strengthening our understanding of the impact of environmental and behavioral variables on the risk of becoming infected with *M. leprae* and potentially developing leprosy. We propose that regular monitoring of the serum anti-NDO-LID antibodies in children and adolescents, and the construction of predictive models for anti-*M. leprae* antibody titers, would be useful among HHC to aid both the early diagnosis of new leprosy cases and track transmission of *M. leprae*. Together, this information can be used to focus control efforts.

## Conclusions

Despite being classified by the WHO as a disease in elimination phase, our results show that, in accordance with the national control program statistics [25], leprosy continues to be a public health issue in several regions in Colombia. This situation points out either failures or a lack of implementation of the eradication strategies proposed by the WHO.

This study has also proven that active *M. leprae* transmission persists in child and adolescent population, mainly in those populations located in vulnerable geographic regions with little presence from government, with armadillo meat consumption traditions, and that are subject to a long exposure to leprosy cases. Therefore, these findings show us that as long as the government does not intervene on the critical socioenvironmental variables distressing juvenile population, leprosy elimination will only be a utopia achieved on the desk of the national control program.

## Additional file

Additional file 1: Validation of multiple regression approach for IgM anti NDO-LID. (DOCX 5547 kb)

## Data Availability

The datasets generated and/or analyzed during the current study are not publicly available but are available from the corresponding author on reasonable request.

## Abbreviations

AR: Andean Region
BCG: Bacillus Calmette-Guerin
CR: Caribbean Region
HHC: Household contact
ICMT: Instituto Colombiano de Medicina Tropical
*M. leprae*: *Mycobacterium leprae*
MB: Multibacillary
NDO-LID: Anti- Natural Octyl Disaccharide-Leprosy IDRI Diagnostic
PB: Paucibacillary
PGL-I: Phenolic glycolipid-I
UAChR: Uraba-Antioquia-Choco Region
