# Supplementary material for: Social and environmental conditions related to Mycobacterium leprae infection in children and adolescents from three leprosy endemic regions of Colombia
Source: BMC Infect Dis. 2019 Jun 13;19:520. doi: 10.1186/s12879-019-4120-2 (PMC6567403; doi:10.1186/s12879-019-4120-2)
Supplement: Supplementary file 1 — Validation of multiple regression approach for IgM anti NDO-LID. (DOCX 5547 kb) [file 12879_2019_4120_MOESM1_ESM.docx]

**Material supplementary**

**Validation of multiple regression approach for IgM anti NDO-LID**

**
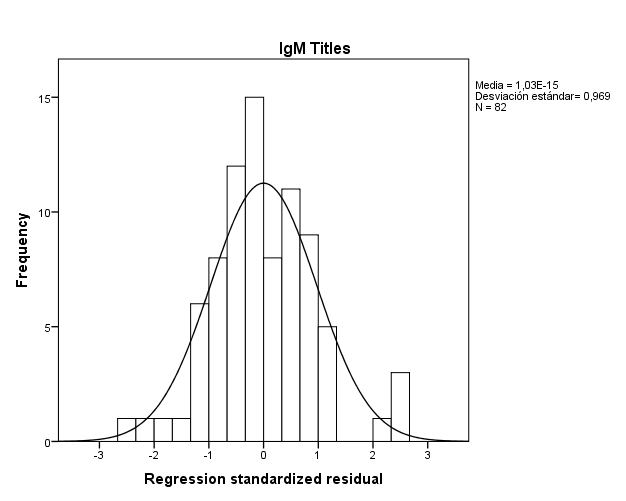
Normal distribution of residuals**

**Linearity (Residuals)**

**Graphic P-P**

**
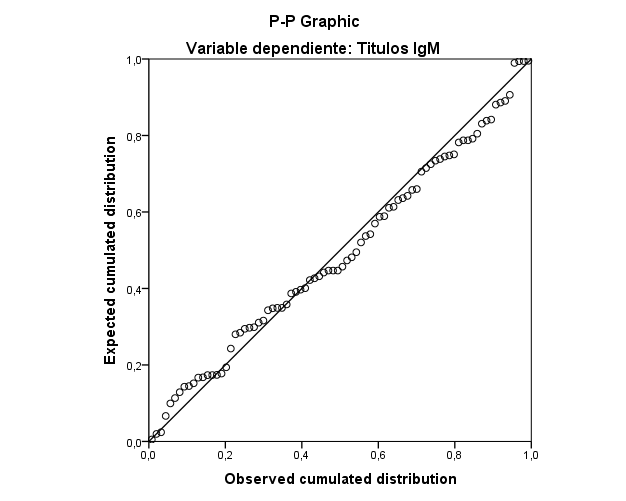
**

**Homoscedastic (Residuals)**


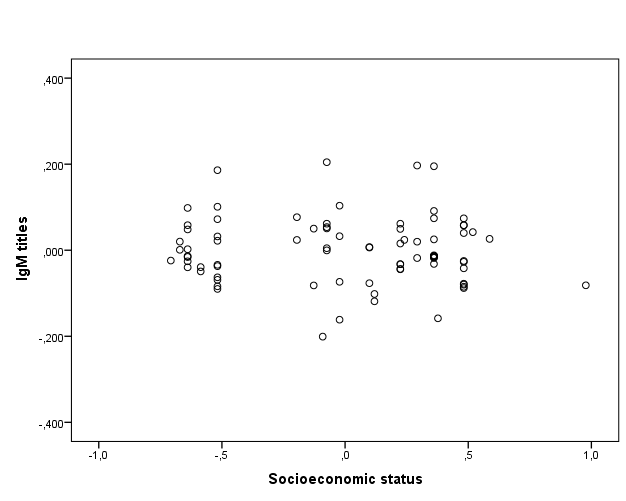
IgM anti NDO-LID titles vs socioeconomic status


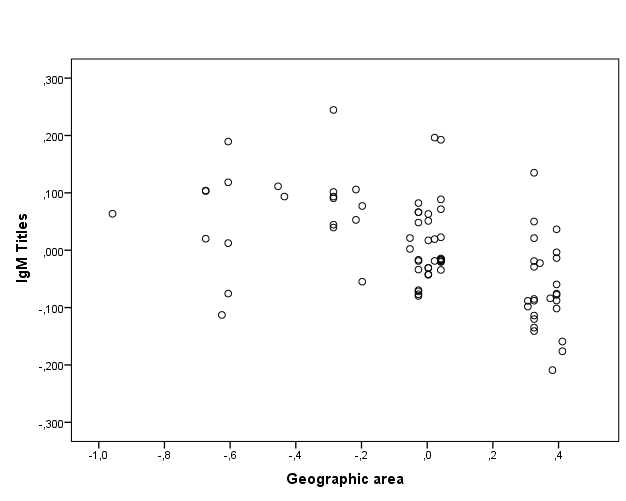
 IgM anti NDO-LID titles vs geographic area


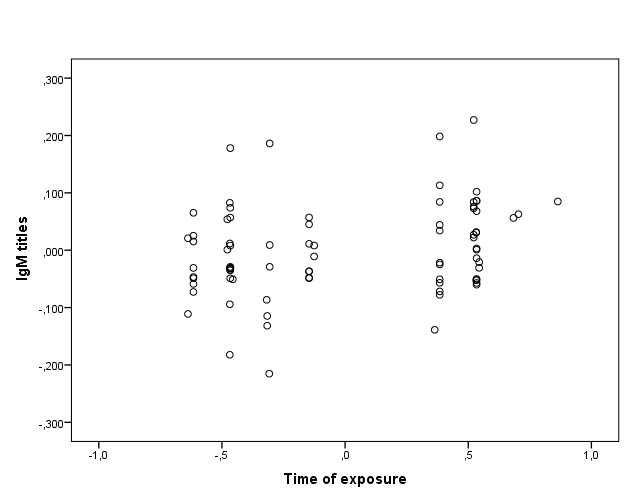
 IgM anti NDO-LID titles vs time of exposure


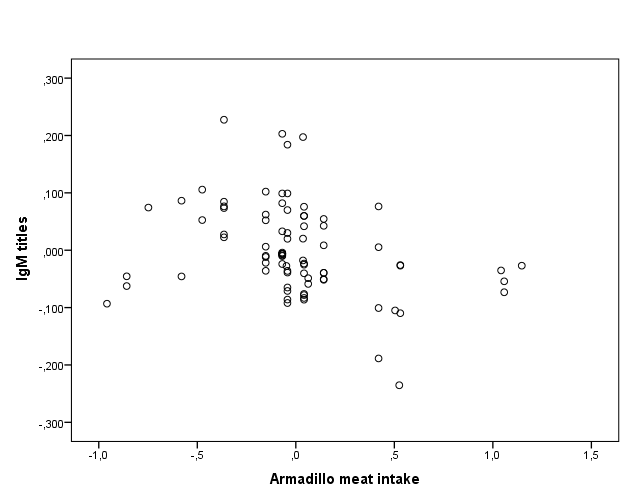
 IgM anti NDO-LID titles vs armadillo meat intake


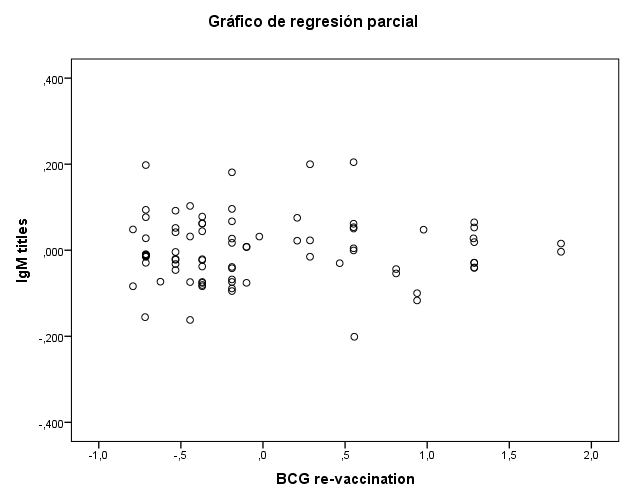
 IgM anti NDO-LID titles vs BCG re-vaccination

**Collinearity**

| Variable | Tolerance | Variance Inflation Factor (VIF) |
| --- | --- | --- |
| Socioeconomic status | 0.733 | 1.36 |
| Geographical area | 0.587 | 1.703 |
| Time of exposure | 0.91 | 1.09 |
| Armadillo meat intake | 0.717 | 1.395 |
| BCG re-vaccination | 0.895 | 1.12 |
